# Characterization of cognitive deficits in spontaneously hypertensive rats, accompanied by brain insulin receptor dysfunction

**DOI:** 10.1186/s40303-015-0012-6

**Published:** 2015-06-04

**Authors:** Edna Grünblatt, Jasmin Bartl, Diana-Iulia Iuhos, Ana Knezovic, Vladimir Trkulja, Peter Riederer, Susanne Walitza, Melita Salkovic-Petrisic

**Affiliations:** University Clinics of Child and Adolescent Psychiatry, University of Zurich, Neumuensterallee 9, 8032 Zurich, Switzerland; Neuroscience Center Zurich, University of Zurich and ETH Zurich, Zurich, Switzerland; University Hospital, Clinic and Policlinic for Psychiatry, Psychosomatic and Psychotherapy, University of Würzburg, Füchsleinstr. 15, D-97080 Würzburg, Germany; Department of Pharmacology and Croatian Institute for Brain Research, School of Medicine, University of Zagreb, Salata 11, 10 000 Zagreb, Croatia; University Clinics of Child and Adolescent Psychiatry, University of Zurich, Wagistrasse 12, CH-8952 Schlieren, Switzerland

**Keywords:** Age, Control strain, Gender, Glycogen synthase-kinase 3β, Insulin resistance, Learning and memory, Spontaneously hypertensive rat

## Abstract

**Background:**

The spontaneously hypertensive rat (SHR) has been used to model changes in the central nervous system associated with cognitive-related disorders. Recent human and animal studies indicate a possible relationship between cognitive deficits, insulin resistance and hypertension. We aimed to investigate whether cognitively impaired SHRs develop central and/or peripheral insulin resistance and how their cognitive performance is influenced by the animal’s sex and age as well as strains used for comparison (Wistar and Wistar-Kyoto/WKY).

**Methods:**

Three and seven-month-old SHR, Wistar, and WKY rats were studied for their cognitive performance using Morris Water Maze (MWM) and Passive Avoidance tests (PAT). Plasma glucose and insulin were obtained after oral glucose tolerance tests. Cerebral cortex, hippocampus, and striatum status of insulin-receptor (IR) β-subunit and glycogen synthase kinase-3β (GSK3β) and their phosphorylated forms were obtained via ELISA.

**Results:**

SHRs performed poorly in MWM and PAT in comparison to both control strains but more pronouncedly compared to WKY. Females performed poorer than males and 7-month-old SHRs had poorer MWM performance than 3-month-old ones. Although plasma glucose levels remained unchanged, plasma insulin levels were significantly increased in the glucose tolerance test in 7-month-old SHRs. SHRs demonstrated reduced expression and increased activity of IRβ-subunit in cerebral cortex, hippocampus, and striatum with different regional changes in phospho/total GSK3β ratio, as compared to WKYs.

**Conclusion:**

Results indicate that cognitive deficits in SHRs are accompanied by both central and peripheral insulin dysfunction, thus allowing for the speculation that SHRs might additionally be considered as a model of insulin resistance-induced type of dementia.

**Electronic supplementary material:**

The online version of this article (doi:10.1186/s40303-015-0012-6) contains supplementary material, which is available to authorized users.

## Background

The spontaneously hypertensive rat (SHR) was inbred from the Wistar-Kyoto rat strain (WKY) [1]. SHRs were initially developed as a model for hypertension but were eventually shown to develop pathologies at the metabolic, behavioral, and cognitive level. The metabolic pathology is manifested as systemic insulin resistance [2]; the behavioral symptoms resemble attention-deficit hyperactivity disorder (ADHD) (hyperactivity, impulsivity, impaired ability to withhold responses, and poorly sustained attention) [3–5]; and the cognitive pathology is manifested as learning and memory deficits [6–8]. Several studies have explored the link between an insulin resistant brain state and cognitive impairment, particularly in dementia of the Alzheimer type as reviewed elsewhere [9–11]. Insulin and insulin receptors (IR) in the brain have been shown to regulate glucose metabolism [12], as expected, but IR also triggers complex signaling pathways in the brain [13–17]. These pathways involve glycogen synthase-kinase-3 (GSK3) inhibition, via protein kinase B (Akt/PKB) [16, 17]. In addition, IR influences the accumulation/degradation of amyloid-β and tau protein, the major neuropathological hallmarks of the memory loss developed in Alzheimer’s disease (AD) [13–15]. We aimed to investigate whether cognitively impaired SHRs develop a central insulin resistance in parallel to the peripheral one, but data published so far on cognitive performance in the SHR model have indicated that measurements were done using different cognitive tests, comparing different control strains, using animals of different ages and not always done using both sexes [3, 18–32]. Since these factors might interfere with cognitive performance in rats, we have additionally explored their possible influence on learning and memory functions in the SHR model.

In brief, following the comparison between different control strains and sexes which showed that female SHRs demonstrate more pronounced cognitive deficits than males, particularly in comparison to the WKY controls, our studies showed that cognitive deficits in female SHRs are accompanied by peripheral and central insulin resistance in 7- but not in 3-month old animals in comparison to WKY controls.

## Methods

### Animals

The experiments were performed on Wistar SHR and 2 normotensive control strains, Wistar-Kyoto (WKY) and Wistar rats, all purchased from Charles River Laboratories (Kisslegg, Germany). Animals were acclimated for 2 weeks before cognitive testing which was performed at the Department of Pharmacology, University of Zagreb School of Medicine (Zagreb, Croatia). Animals consumed standardized food pellets and water ad libitum.

### Ethics

All procedures were performed under the guidance of the *Principles of Laboratory Animal Care* (NIH Publication No. 80-23, revised in 1996) and in accordance with the European Communities Council Directive of 24 November 1986 (86/609/EEC) and the Croatian Act on Animal Welfare (NN 135/2006). All experiments were approved by the University of Zagreb School of Medicine (Licence No.04-1343-2006).

### Experimental design

Three different experiments were performed in vivo to explore the effect of (1) control rat strain, with 12-week male SHR, WKY and Wistar rats, (2) sex, with 12-week-old female and male SHR and WKY rats, and (3) age, with 12- and 28-week-old (i.e. 3- and 7-month-old) female SHR and WKY rats. Animal age in the third experiment corresponded to young adult and older, “middle-aged” humans [33] as well as represented the periods of rising blood pressure (3 months) and sustained/chronic hypertension (7 months) [34, 35]. There were 10 animals per group in all experiments.

### Cognitive testing

#### Morris Water Maze (MWM) swimming test

The MWM tested learning ability and spatial memory as previously described and used in our experiments [36, 37] (details provided in the Additional file 1). The time needed to find the platform (seconds) and the number of errors (incorrect entries into quadrants with no platform) were recorded in training trials during 4 consecutive days while the time spent searching for the platform after entering quadrant IV and number of errors were recorded in the probe trial that followed.

#### Passive Avoidance test (PAT)

The step-through PAT was performed two days after finishing the MWM test and exploited a fear-motivated tendency of a rat to escape from an illuminated area into a dark area as previously described and used in our experiments [36, 37]. Latency time (seconds) before entering the dark area was recorded on the third testing day. A more detailed PAT method is provided in the Additional file 1.

### Oral glucose tolerance test

The OGTT was performed after the rats were under deep chloral hydrate (300 mg/kg i.p.) anesthesia, in accordance with the ethical requirements for laboratory animal procedures of the Medical School University of Zagreb. Blood samples were sequentially collected from the tail vein (50 μL; between 8 and 12 a.m.) for plasma glucose measurements at baseline, 30, and 60 min after the challenge. The post-OGTT cut-off time of 60 min corresponded to the duration of anesthesia from a single chloral hydrate dose. Animals were sacrificed after the last blood withdrawal still in deep anesthesia, 2 days after last cognitive testing.

### Preparation of brain regions

Macrodissection of three brain regions (frontal cortex, striatum, and hippocampus) was performed on a cold plate (4 °C) by a standard procedure and according to the rat brain atlas [38]. The brain samples were snap frozen and stored at -80 °C until further analysis.

#### Brain homogenates

Frozen brain tissue from each rat was weighed, and a 4-fold concentration of RIPA buffer (Sigma-Aldrich, Schelldorf, Germany) was added with protease and phosphatase inhibitors (Sigma-Aldrich, Schelldorf, Germany). Samples were homogenized via ultrasonic disruption (15 % amplitude, 10 s) at 4 °C. After homogenization, the probes were centrifuged at 44,000 × *g* at 4 °C for 10 min. Clear supernatant (which contained proteins) was collected into a fresh tube for further analysis.

#### Protein concentration

For each sample, protein concentrations were evaluated with the standard Bradford method for protein measurement [39].

### Biochemistry

Plasma glucose concentrations were measured with a commercial kit (Glucose-PAP Test, Herbos Diagnostics) and the glucose oxidase method. Plasma insulin levels were assessed with a commercial kit for an Enzyme-Linked Immunosorbent Assay (ELISA; Crystal Chem Inc, IL, USA; Cat. No. 90060), which was more sensitive for quantitative analysis (sensitivity <0.8 units/ml) than a Western Blot assay. ELISAs were used to evaluate brain expression levels of the insulin receptor (IR) β-subunit (total), the two forms of the phosphorylated-IRβ subunit, pTyr^1162/1163^ and pTyr^1158^ (Calbiochem, Darmstadt, Germany; CBA039, CBA038, and Invitrogen GmbH, Darmstadt, Germany; KHR9121, respectively), the glycogen synthase-kinase (GSK)-3 β-subunit (total), and the phosphorylated form of GSK-3β, pSer^9^ (Calbiochem, Darmstadt, Germany; CBA068 and CBA069, respectively). All ELISAs were performed as recommended by the manufacturer. In the ELISA tests, brain homogenates were run in parallel with standards for concentration evaluations.

### Statistical analysis

The statistical analyses of all data were performed with StatView for Windows (SAS Institute Inc., Cary, NC, USA; Version 9.3). Data from the MWM training trials were analyzed by fitting general(ized) linear mixed models (Poisson link for the number of errors, normal for escape latency. Instead of adjustment for multiple comparisons, which we considered too conservative, main effects (across all days or both genders/strains) of strain and gender are given with 95 % confidence interval (CI); contrasts arising from interactions with up to 4 cross-products (strain*gender across all days, strain*age across all days) are given with 97.5 % CI; contrasts arising from strain*(sex or age)*day interaction are given with 99 % CI (the significance level *p* < 0.05, <0.025 or <0.01, respectively). Additionally, the multifactorial analysis of variance (MANOVA), followed by the analysis of Post-Hoc Scheffé test, and the Mann–Whitney *U*-test, was used. The significance level was set to p <0.05.

## Results

### Cognitive deficits

After demonstrating that SHR rats performed poorly in MWM training trials regardless of the control strain (WKY or Wistar) (*p* < 0.05), but that the difference was more pronounced compared to WKY (*p* < 0.05), WKY rats were used as a control in further experiments, which consistently showed that SHRs performed worse regardless of sex (*p* < 0.05) or age (*p* < 0.05), as presented in Additional file 2: Figure S2 & Additional file 3: Figure S3.

**Fig. 1:**
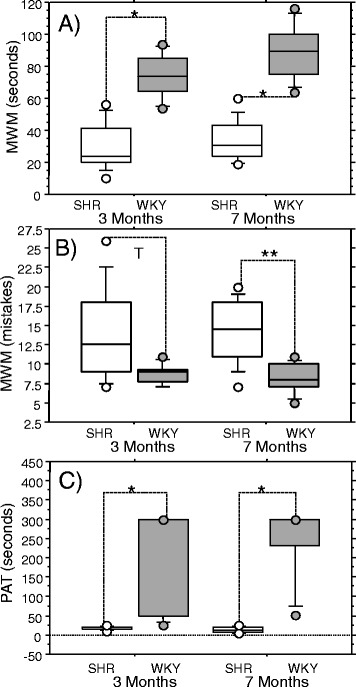
Results of Morris Water Maze probe trial and Passive Avoidance test in spontaneously hypertensive rats. Results are shown for 3- and 7-month-old female (**a**, **b**, **c**) animals. Morris Water Maze (MWM) test–probe trial results: **a** time spent swimming within the targeted quadrant; **b** number of entries into the wrong quadrant (mistakes). Passive avoidance test (PAT) results: **c** latency time. Line in the boxes represent the median, whiskers are the 10 and 90 % percentile. Female spontaneously hypertensive rats (SHR) = *White box*; Female Wistar Kyoto control rats (WKY) = *Grey box*. Significant differences were based on the ANOVA Post-Hoc Scheffé; *p < 0.0001 vs. controls; **p < 0.05; T (tendency) 0.05 < *p* < 0.1; *n* = 10

As we could show that the most pronounce differences in cognitive deficits in training trials were in female SHR compared to female WKY (*p* < 0.05), and in some extent due to age (*p* < 0.05) (Additional file 3: Figure S3), the cognitive performance of 3- and 7-month old female SHR and WKY rats was further recorded in the MWM probe trials. Both SHR age groups demonstrated significant deficits in spatial memory; they spent less time swimming in the quadrant from which the platform has been removed (*p* < 0.0001; Fig. 1a) and made more errors than controls (7-month-old SHRs, *p* < 0.05; 3-month-old SHRs, *p* = 0.06; Fig. 1b). Female SHRs of both ages also showed marked deficits in fear-motivated memory in the PAT. They demonstrated much lower post-shock latency times than WKY controls (*p* < 0.0001, Fig. 1c), with no age-related effect being detected.

### Insulin receptor signaling in the brain

Based on the results of cognitive testing, female SHRs were further used to investigate whether cognitive deficits are accompanied by alterations of insulin signaling in the brain, when compared to WKY controls.

#### Brain IRβ expression levels

**Fig. 2:**
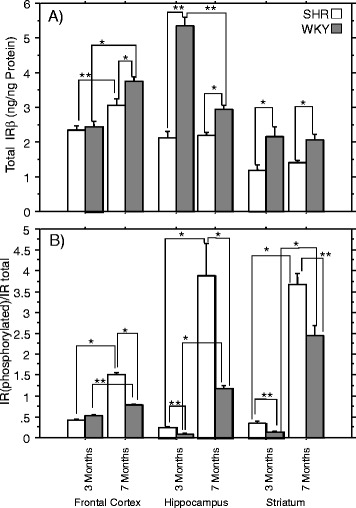
Brain insulin receptor-β-subunit levels and activity in spontaneously hypertensive and Wistar Kyoto control rats. (**a**) Insulin receptor (IR)β-subunit levels and (**b**) the ratio of IRβ phosphorylated (pTyr^1158^ and pTyr^1162/1163^)/total IRβ are shown for different brain regions of 3- and 7-month-old spontaneously hypertensive rats (SHRs = *Whit*e) and Wistar Kyoto control rats (WKY = *Grey*). Columns represent the mean ± SEM Significant differences were based on the ANOVA Post-Hoc Scheffé test; *p < 0.0001; **p < 0.05; *n* = 10

SHRs had, in general, lower total IRβ subunit levels than WKY rats in all brain regions, particularly in the hippocampus and striatum (Fig. 2a) but showed age-dependent increment in IRβ level in the frontal cortex only (*p* < 0.05, Fig. 2a) in contrast to region-dependent variability in the total IRβ levels with aging in WKY controls (frontal cortex–increased /*p* < 0.05/, hippocampus–decreased /*p* < 0.05/, striatum–unchanged, in 7- compared to 3-month-old animals, respectively) (Fig. 2a).

#### Brain IR activity

IR activity expressed as the ratio of phosphorylated IR (pTyr^1162/1163^ and pTyr^1158^) to total IRβ (higher ratio indicating higher IR activity) was significantly increased in all investigated brain regions in 7- compared to the 3-month-old animals in both SHRs and WKYs but the magnitude of age-dependent increment was larger in SHRs than in WKYs (Fig. 2b). Compared to WKYs, SHRs had significantly higher IR activity at both ages in the hippocampus and striatum and at 7-month of age in the frontal cortex (*p* < 0.05; Fig. 2b).

#### Brain GSK3β expression levels and activity

**Fig. 3:**
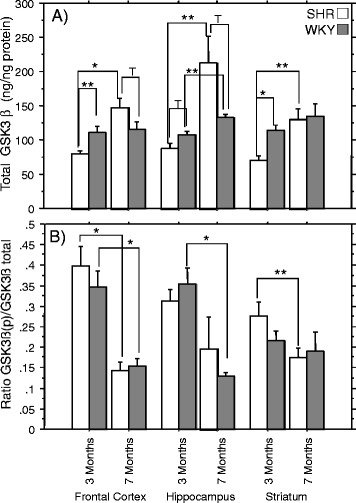
Brain glycogen synthase-kinase-3 β-subunit levels and activity in spontaneously hypertensive and Wistar Kyoto control rats. (**a**) Glycogen synthase-kinase-3 β-subunit (GSK3β) levels and (**b**) the ratio of GSK3β phosphorylated (pSer^9^)/GSK3β total are shown for different brain regions of 3- and 7-month-old spontaneously hypertensive rats (SHRs = *White*) and Wistar Kyoto control rats (WKY = *Grey*). Columns represent the mean ± SEM. Significant differences were based on the ANOVA Post-Hoc Scheffé test; *p < 0.005; **p < 0.05; 0.05 < T < 0.1; *n* = 10

Total GSK3β level in the frontal cortex (*p* < 0.0005), hippocampus (*p* < 0.0001) and striatum (*p* < 0.005) was significantly lower in 3- compared to 7-month-old SHRs (Fig. 3a) while WKYs expressed such age-dependent difference only in the hippocampus (*p* < 0.05; Fig. 3a). Since GSK3β activity is regulated mainly via phosphorylation of the serine-9 residue, we determined its relative activity expressed as the ratio of the phosphorylated (inactive) to total GSK3β form (low ratio indirectly suggests high GSK3β activity). Age-dependent tendency of decrement in this ratio (increased GSK3β activity) was observed in both SHRs and WKYs with significance reached only in 7-month-old animals but with some regional specificity; SHRs - frontal cortex and striatum (*p* < 0.05), WKY - frontal cortex (*p* < 0.005) and hippocampus (*p* < 0.0001) (Fig. 3b).

### Plasma glucose and insulin levels

**Fig. 4:**
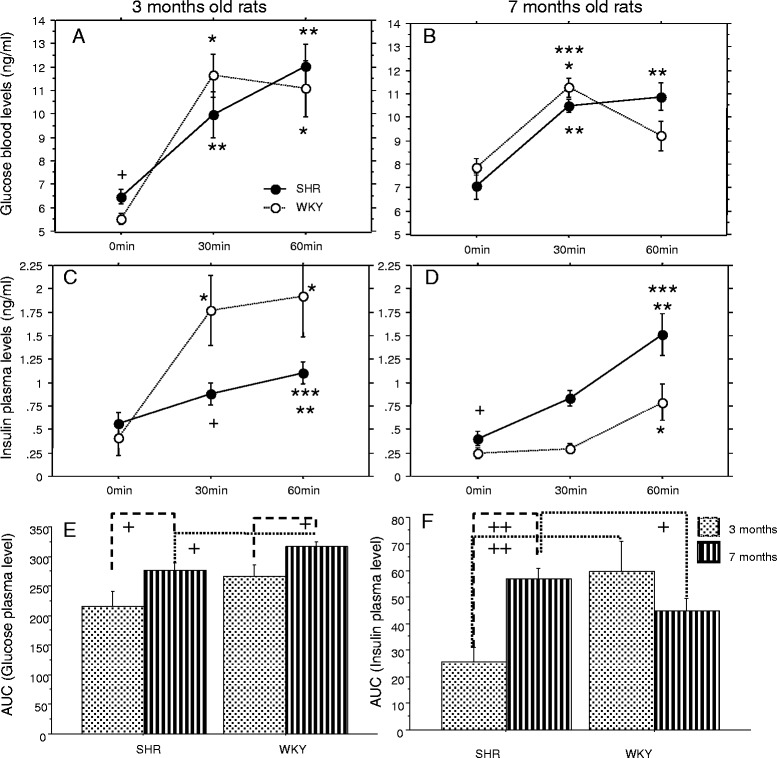
Glucose challenge results for 3- and 7-month-old rats. Plasma glucose and insulin levels were measured at baseline, 30 min and 60 min after the glucose tolerance test. **a** Plasma glucose levels in 3-month-old spontaneously hypertensive rats (SHR, *continuous line* + *black circle*) and Wistar Kyoto controls (WKY, *dashed line* + *white circle*); **b** plasma glucose levels in 7-month-old SHRs and controls; **c** plasma insulin levels in 3-month-old SHRs and controls; **d** plasma insulin levels in 7-month-old SHRs and controls; **e**-**f** Area under the curve (AUC) of glucose and insulin values from the oral glucose tolerance test (OGTT) of SHR and WKY in 3- and 7-months old animals (*points & lines bars*, respectively). *Bars* represent the mean ± SEM. Significant differences were based on the ANOVA Post-Hoc Scheffé; *p < 0.05 vs. baseline control; **p < 0.05 vs. baseline SHR; ***p < 0.05 vs. 60 min control; + *p* < 0.05 vs. 30 min control; (e & f) + *p* < 0.05; ++ *p* < 0.01; *n* = 10

Both 3- and 7-month-old SHRs showed a significant increase in plasma glucose after 30 and 60 min compared to the baseline levels in the oral glucose tolerance test (OGTT; Fig. 4a and b). A similar time-pattern effect was detected in WKYs with the exception of the 60 min time-point in 7-month-old WKYs when glucose levels were not significantly different from the respective baseline values (Fig. 4a and b). In contrast, the time-course of the plasma insulin levels showed opposite effects in the 3- and 7-month-old rats. In 3-month-old animals, SHRs showed only modest increase in plasma insulin with borderline significant differences at 60 min compared to the baseline levels, while insulin plasma levels in WKYs were significantly increased at both time-points compared to their baseline level (Fig. 4c). In the 7-month-old animals, both the SHRs and WKYs showed significant increases in plasma insulin at the 60 min time-point only, compared to their baseline levels (Fig. 4d). Analysis of the area under the curve (AUC) values of plasma glucose and insulin (Fig. 4e-f) in SHRs and WKYs of both ages pointed to an age-dependent disruption of glucose metabolism for both groups. But to the contrary, plasma insulin AUC was significantly increased with age in the SHR animals, while no significant age-dependent difference was observed in WKY controls.

## Discussion

Consistent with previous reports [7, 40–42], this study confirmed that the SHR model develops significant memory and learning deficits, which was a prerequisite for the study’s main aim: to explore whether cognitive deficits in SHRs are accompanied by peripheral/central metabolic abnormalities. Considering literature data discussing that selection of corresponding control group (rat strain and usage of inbred, e.g. Wistar-Kyoto/WKY/, or outbred, e.g. Wistar, Sprague–Dawley/SD/animals) as well as age and sex might affect the results of cognitive testing [23, 25, 43], we investigated the cognitive profiles of the optimal control strain and SHR gender and identified those which demonstrated most pronounced difference between the control and SHR group, to be used in exploring the metabolic changes in this model.

Our experiments (Additional file 2: Figure S2 & Additional file 3: Figure S3) provided evidence that the SHR model performed poorly compared to control regardless sex, but females performed worse than males. Some literature data have suggested that hyperactivity behavior might influence the cognitive performance of SHRs and that locomotor activity (distraction) could be a confounding factor in spatial memory tasks [24], for example, in hyperactive female SHRs [32]. Although increased locomotor activity might act as a distraction in achieving a goal, hyperactivity in a cognitively normal rat might also lead to acheiving this goal in a more focused, direct way than in a rat with normal locomotor activity (which was not the case in our experiments). The present data (Additional file 2: Figure S2), however, strongly suggest that the observed deficit manifested as an increased number of errors was not due to hyperactivity: compared to controls, SHR rats made more errors (Additional file 2: Figure S2a, d) and had longer latency times (Additional file 2: Figure S2b, e), but time spent *per* one error was the same in all strains (Additional file 2: Figure S2c), and with adjustment for the number of errors, latency times did not differ between the SHR and control strains (Additional file 2: Figure S2F). Had hyperactivity (as a distraction factor) been the cause of the increased error rate, one would have expected to see reduced latency times per one error (e.g., faster swimming due to locomotor hyperactivity), or extended latency times per error (e.g., “non-focused” swimming due to hyperactivity), but not the same latency time per error. Since cognitive deficits in our experiments with cognitively more impaired female SHRs were more pronounced in comparison to WKY than to Wistar animals, female SHRs and WKYs were used for further exploration of metabolic abnormalities. WKY and Wistar strains have been frequently used as a control for memory testing in SHRs [22, 23, 25, 43, 44], but usually not in the same experiment with the exemption of the work of Gattu et al. [45, 46] which showed cognitive impairment of 1- and 3-month-old male SHRs compared to both these control strains.

A growing body of evidence points to the association of cognitive deficits and insulin resistance in humans and in animals [47–49], an issue that has been poorly explored in the SHR model; a presence of systemic insulin resistance in SHRs has been manifested by a plasma insulin response in 7 week-old animals (sex not specified) [2], and indirectly by a reduced insulin-stimulated glucose transporter bioavailability in adipocytes [50], while an unchanged expression of insulin receptor in the brain of 3-month-old male SHRs was reported by Yang et al. [51]. Our results indicate that the plasma insulin response to an oral glucose challenge is altered in SHRs compared to WKY but that the direction of change depends on a respective time-point of its measurement; lower plasma insulin response in 3-month (AUC = 25/SHR vs. 60/WKY) and greater in 7-month-old female SHRs (AUC = 58/SHR vs. 45/WKY) despite the similarity in the total serum glucose response in SHRs and WKYs. Such a difference in insulin AUC after OGTT was not detected in a recent study investigating various combinations of metabolic syndrome and AD markers in male SHR animals [51], indicating that a possible gender influence in this model might play a role not only in cognition but also in insulin response. Additionally, it could not be excluded that this time-dependent insulin response might correlate with an observed tendency for age-dependent worsening of learning and memory functions in female SHRs, indirectly also reflecting a difference in metabolic response between early (3-month-old) and chronic (7-month-old) hypertension.

Although several studies have suggested that learning and memory deficits observed in SHR animals were independent of hypertension [30, 40, 52], others did find correlations between hypertension and cognitive deficits [5, 6, 41, 53]. Having in mind that aging, female gender, and hypertension are considered risk factors for dementia, particularly of the Alzheimer’s type (see meta-analysis in www.alzrisk.org and [54–57], usage of aged SHR animals with reduced cognitive performance might be worth considering as a possible model of a certain type of dementia [5, 58].

Several findings in SHR animals point to their susceptibility to metabolic alterations in the brain. For example, recent findings of Ritz et al. [59] showed that gene expression alterations in the cortex of 2-month-old (pre-hypertension) and 9-month-old SHRs (compared to WKY) pointed to a vulnerability to high-energy requirements in these animal models. Furthermore, studies of cerebral glucose utilization in SHR compared to WKY were found to be lower at aged animals (around 6 months) [60, 61] while not different in younger ones (12 weeks only) [62, 63]. Gene expression profiling in cell cultures originating from the brain stems of SHR and WKY animals revealed significantly enriched genes belonging to molecular pathways of the ATPase activity [64], further supporting the involvement of energy metabolism dysfunction in this model.

Glucose/energy hypometabolism in the brain might be a consequence of an insulin resistant brain state [65, 66], which seems to be involved in cognitive decline, as suggested in particular in AD and its animal models [9–11, 47, 67]. This hypothesis has been recently discussed for AD patients pointing to the pathophysiological importance of the vicious cycle of glucose/energy demand, vascular cognitive impairment, dementia, and aging, suggesting that an understanding of how vascular and metabolic factors interfere with a progressive loss of functional neuronal networks becomes essential for developing efficient drugs to prevent cognitive decline in the elderly [68]. Keeping in mind that glucose metabolism in the brain is under control of brain IR [12], our findings of dysfunction in brain IR and its downstream signaling cascade might contribute to the pathophysiological mechanisms of the cerebral glucose hypometabolism in SHRs reported in the literature [60]. We generally found lower total levels of the IRβ subunit in SHRs compared to WKYs in all brain regions investigated, regardless of a rat’s age. This finding might indicate that SHRs have a general deficit in brain insulin signaling pathways. Furthermore, we observed an increased IR activity in the 7-month-old SHRs, measured as the ratio of phosphorylated to total IRβ subunits, which might represent a compensatory mechanism covering for reduced IRβ level. Yang and colleagues [51] could not find difference in IR, which, however, was measured in the whole brain (and not in specific brain regions as in our experiment) of young male SHR animals compared to WKY. Our findings demonstrated that GSK3β activity (indirectly measured by p/total GSK3β ratio), which is down-stream to IR signaling and is linked to hyperphosphorylation of tau-protein [69], increased with aging in the cerebral cortex of both the SHR and WKY group, while in the hippocampus it was significantly increased only in 7-month-old female SHRs compared to corresponding WKYs. In recent years, the over-activation of GSK3β in the brain has been reported to be involved in the pathophysiology of AD and of type-2 diabetes mellitus [70].

These results further support the hypothesis that the SHR model develops insulin resistance in the brain, manifested both at the level of IR protein/activity and its downstream signaling pathway, as well at the periphery. Moreover, our results obtained in the same pool of animals demonstrate for the first time the co-existence of hypertension with peripheral and central insulin resistance and cognitive deficits in SHR model.

## Conclusions

This study provides the first combined evidence that cognitive deficits in the SHR model are accompanied by insulin-signaling dysfunction in the brain in parallel to the existence of peripheral insulin resistance. Factors like rat strain used for comparison, SHRs age and/or hypertension duration as well as sex should be considered in the interpretation of cognitive and metabolic performance of the SHR model. Further studies are required to address the question of a possible causal relationship between these metabolic and cognitive impairments in SHRs and accordingly explore whether SHRs might be also considered as a model of insulin resistance-induced type of dementia, in addition to being a model of hypertension and ADHD.

## Availability of supporting data

The three data sets supporting the results of this article are included within the article (and its additional files). 1) Additional file 1: Detailed cognitive testing methods; 2) Additional file 2: Figure S2: Comparison of the cognitive performance in the Morris Water Maze (MWM) training trials; 3) Additional file 3: Figure S3: The effect of sex and age on the cognitive performance of spontaneously hypertensive rats (SHR).

## Additional files

Additional file 1:
**Detailed cognitive testing methods.**
Additional file 2:
**Comparison of the cognitive performance in the Morris Water Maze (MWM) training trials.**
Additional file 3:
**The effect of sex and age on the cognitive performance of spontaneously hypertensive rats (SHR).**

## Abbreviations

AD: Alzheimer’s disease
ADHD: Attention-deficit hyperactivity disorder
AUC: Area under the curve
CBF: Cerebral blood flow
CI: Confidence interval
CNS: Central nervous system
ELISA: Enzyme-Linked Immunosorbent Assay
GSK3: Glycogen synthase-kinase-3
IR: Insulin receptor
IRβ: IR β-subunit
MWM: Morris Water Maze
MANOVA: Multifactorial analysis of variance
OGTT: Oral glucose tolerance test
PAT: Passive Avoidance Test
Akt/PKB: Protein kinase B
SHR: Spontaneously hypertensive rat
SD: Sprague–Dawley
STZ: Streptozotocin
WKY: Wistar-Kyoto rat strain
