# Supplementary material for: Characterization of cognitive deficits in spontaneously hypertensive rats, accompanied by brain insulin receptor dysfunction
Source: J Mol Psychiatry. 2015 Jun 4;3(1):6. doi: 10.1186/s40303-015-0012-6 (PMC4479234; doi:10.1186/s40303-015-0012-6)
Supplement: Additional file 1: — Detailed cognitive testing methods. [file 40303_2015_12_MOESM1_ESM.docx]

**Additional file 1: Detailed cognitive testing methods**

***Cognitive testing***

After being transported from the Charles River Laboratories to the Department of Pharmacology, Medical School University of Zagreb, animals were put under quarantine for two weeks before testing of learning and memory functions.

*1. Morris Water Maze (MWM) Swimming Test*

The MWM tested learning ability and spatial memory as previously described [1, 2]. Two days before the experimental trials, rats were allowed to adapt to the experimental environment and their behavioural activity was observed. On the first adaptation day, animals were subjected to 1 min free swimming in a pool (150 × 60 cm, 50 cm deep), filled with clear water at a temperature of 25±1 °C. On the next adaptation day, the rats were allowed to swim freely, but the pool was divided into four quadrants (I–IV).

The experimental trials were performed on Days 1 to 4 during which rats first underwent ‘training trials”, where they could escape from water when they found a hidden, clear glass platform, submerged 2 cm below the water surface in quadrant IV. Rats were allowed to remain on the platform to memorize its location for 15 s. One trial consisted of three starts, each from a different quadrant (I–III), separated by a 1-min rest period. Three consecutive trials were performed *per* day, separated by a 30-min rest period. Such a design of MWM is in line with published protocols suggesting that performance of MWM test may vary among different laboratories in number of swim starts per trial or number of trials per day and/or number of testing days [3]. The time needed to find the platform (seconds) and the number of mistakes (incorrect entries into quadrants with no platform) were recorded in each trial during the 4 days of testing learning ability. After the last training trial on Day 4, the rats underwent the ‘probe trial’ following a 30 min break, to test for retention of spatial memory. In the probe trial, rats performed 3 starts from quadrants I–III, but the platform had been removed from the pool. The time spent searching for the platform after entering quadrant IV and the number of mistakes was recorded for the probe trial. The probe trial ended after 1 min.

We anticipated that, compared to the control rats, rats with reduced learning and memory functions would have difficulties in remembering the location of the platform in the training trials, and consequently, they would make more errors and need more time to find the platform. In addition, in the probe trial, rats with learning and memory deficits were expected to spend less time swimming in the quadrant that previously held the platform and make more entries into quadrants that had not previously held the platform (a higher number of mistakes).

***2. Passive Avoidance Test (PAT)***

The PAT was performed two days after finishing the MWM test. The PAT exploits the tendency of a rat to escape from an illuminated area into a dark area. The step-through passive avoidance system (Ugo Basile, Comerio, Italy, Cat. No. 7570) consisted of a box divided into two (illuminated and dark) compartments with a sliding door and a control unit that incorporated an electric shock device. On the first day of the experiment, animals (one at a time) were allowed to explore the instrument freely. On the second day, the shock device was activated in the dark compartment to deliver a low intensity electro-shock (0.3-0.5 mA/2 s) through a floor grid when the animal entered the dark compartment. On the third day, the shock device was turned off in the dark compartment and activated in the illuminated compartment, with a delay. When the animal remained in the illuminated compartment for more than 5 min, the animal received a shock until stepping into the dark compartment. The step-through PAT was used to test fear-motivated memory. Compared to the control rats with no memory deficits, those with memory deficits were expected to have no retention of the memory that they received an electro-shock the day before; thus, on the third day, they were expected to enter the dark compartment more readily, and thus, demonstrate shorter latency times than controls.

***References***

1. Salkovic-Petrisic M, Osmanovic-Barilar J, Knezovic A, Hoyer S, Mosetter K, Reutter W. Long-term oral galactose treatment prevents cognitive deficits in male Wistar rats treated intracerebroventricularly with streptozotocin. Neuropharmacology. 2014;77:68-80. doi:10.1016/j.neuropharm.2013.09.002.

2. Salkovic-Petrisic M, Tribl F, Schmidt M, Hoyer S, Riederer P. Alzheimer-like changes in protein kinase B and glycogen synthase kinase-3 in rat frontal cortex and hippocampus after damage to the insulin signalling pathway. J Neurochem. 2006;96(4):1005-15.

3. Vorhees CV, Williams MT. Morris water maze: procedures for assessing spatial and related forms of learning and memory. Nat Protoc. 2006;1(2):848-58. doi:nprot.2006.116 [pii]

10.1038/nprot.2006.116.
