# Supplementary material for: Characterization of cognitive deficits in spontaneously hypertensive rats, accompanied by brain insulin receptor dysfunction
Source: J Mol Psychiatry. 2015 Jun 4;3(1):6. doi: 10.1186/s40303-015-0012-6 (PMC4479234; doi:10.1186/s40303-015-0012-6)
Supplement: Additional file 2: — Comparison of the cognitive performance in the Morris Water Maze (MWM) training trials. [file 40303_2015_12_MOESM2_ESM.docx]

**Additional file 2: Comparison of the cognitive performance in the Morris Water Maze (MWM) training trials.**


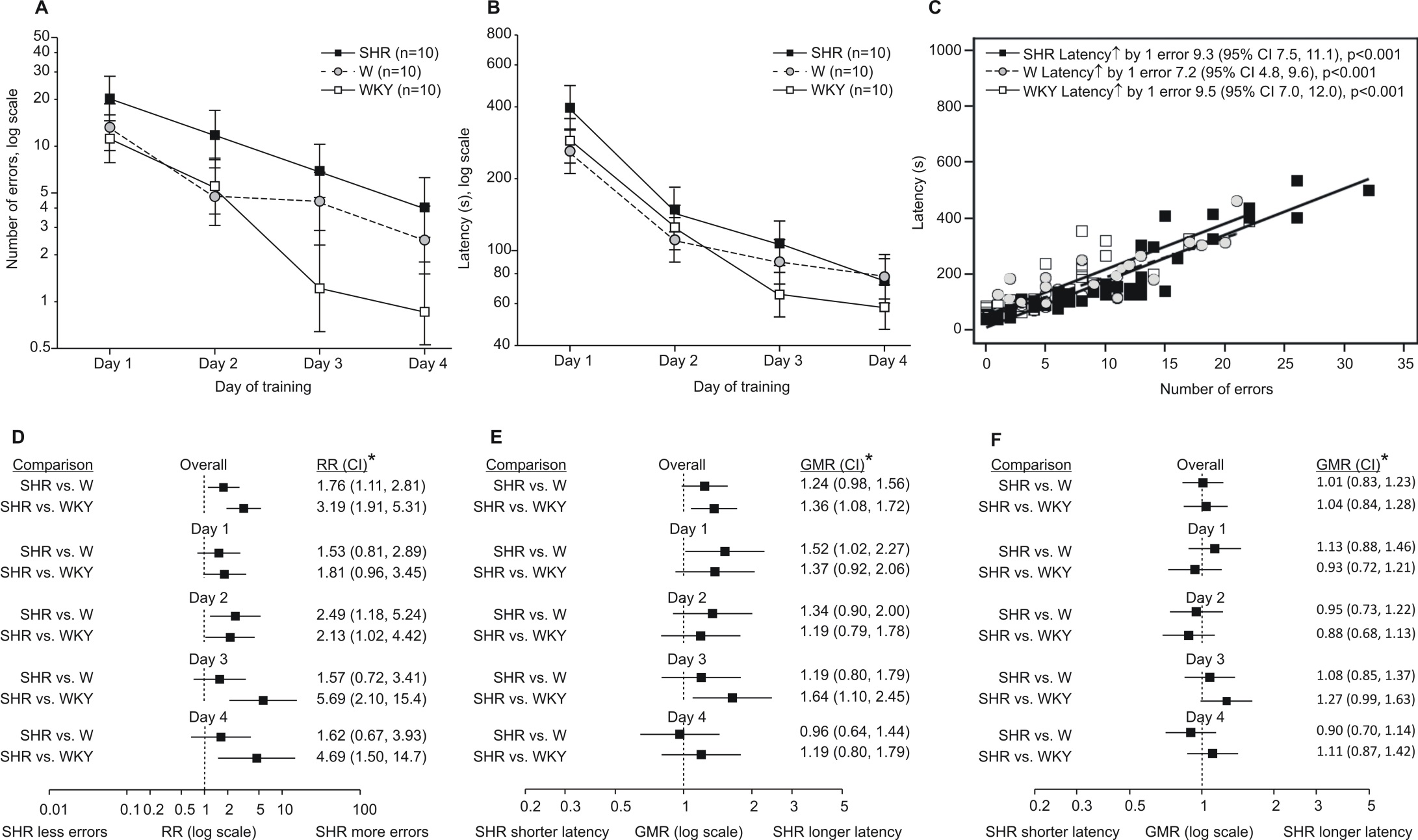


**Figure S2:** *Comparison of the cognitive performance in the Morris Water Maze (MWM) training trials.* The comparison was conducted between the control Wistar (W) and Wistar-Kyoto (WKY), and spontaneously hypertensive rats (SHR). Twelve-week old (3-months) male SHRs perform worse than either age- and sex-matched W or WKY control rats. Animals were submitted to MWM training cycles over 4 consecutive days. (A) Geometric means (95% confidence intervals) of cumulative number of errors (9 runs per animal) over time. A generalized linear mixed model (Poisson) was fitted to data with fixed factors strain, time and strain*time interaction, and time as a random (repeated) factor. (B) Geometric means (95% confidence intervals) of cumulative latency time (seconds) (9 runs per animal) over time. A general linear mixed model was fitted to data with fixed factors strain, time and strain*time interaction, and time as a random (repeated) factor. (C) Relationship between number of errors and latency times for the three strains (all data over four testing days). A general linear mixed model was fitted to latency time data with fixed factors strain, number of errors and number of errors*strain interaction, and time (testing day) as random (repeated) factor.(D) Differences in error numbers between SHR and control animals derived from the model depicted in (A). Differences (effects) are presented as relative risks (RR). (E) Differences between SHR and control animals in latency times derived from the model depicted in (B). Differences (effects) are presented as geometric means ratios (GMR). (F) Differences between SHR and control animals in latency times with adjustment for the number of errors [derived from the model depicted in (B) with “number of errors” as an addition independent]. Differences (effects) are presented as geometric means ratios (GMR).

*Instead of adjustment for multiple comparisons, which we considered too conservative, overall effects of SHR are given with 97.5% CI. For the contrasts from the strain*time interaction, 99% CI are displayed. Hence, were the risk or geometric means ratio is entirely below or above unity, p<0.025 or <0.01.
