# Supplementary material for: Characterization of cognitive deficits in spontaneously hypertensive rats, accompanied by brain insulin receptor dysfunction
Source: J Mol Psychiatry. 2015 Jun 4;3(1):6. doi: 10.1186/s40303-015-0012-6 (PMC4479234; doi:10.1186/s40303-015-0012-6)
Supplement: Additional file 3: — The effect of sex and age on the cognitive performance of spontaneously hypertensive rats (SHR). [file 40303_2015_12_MOESM3_ESM.docx]

**Additional file 3: The effect of sex and age on the cognitive performance of spontaneously hypertensive rats (SHR).**


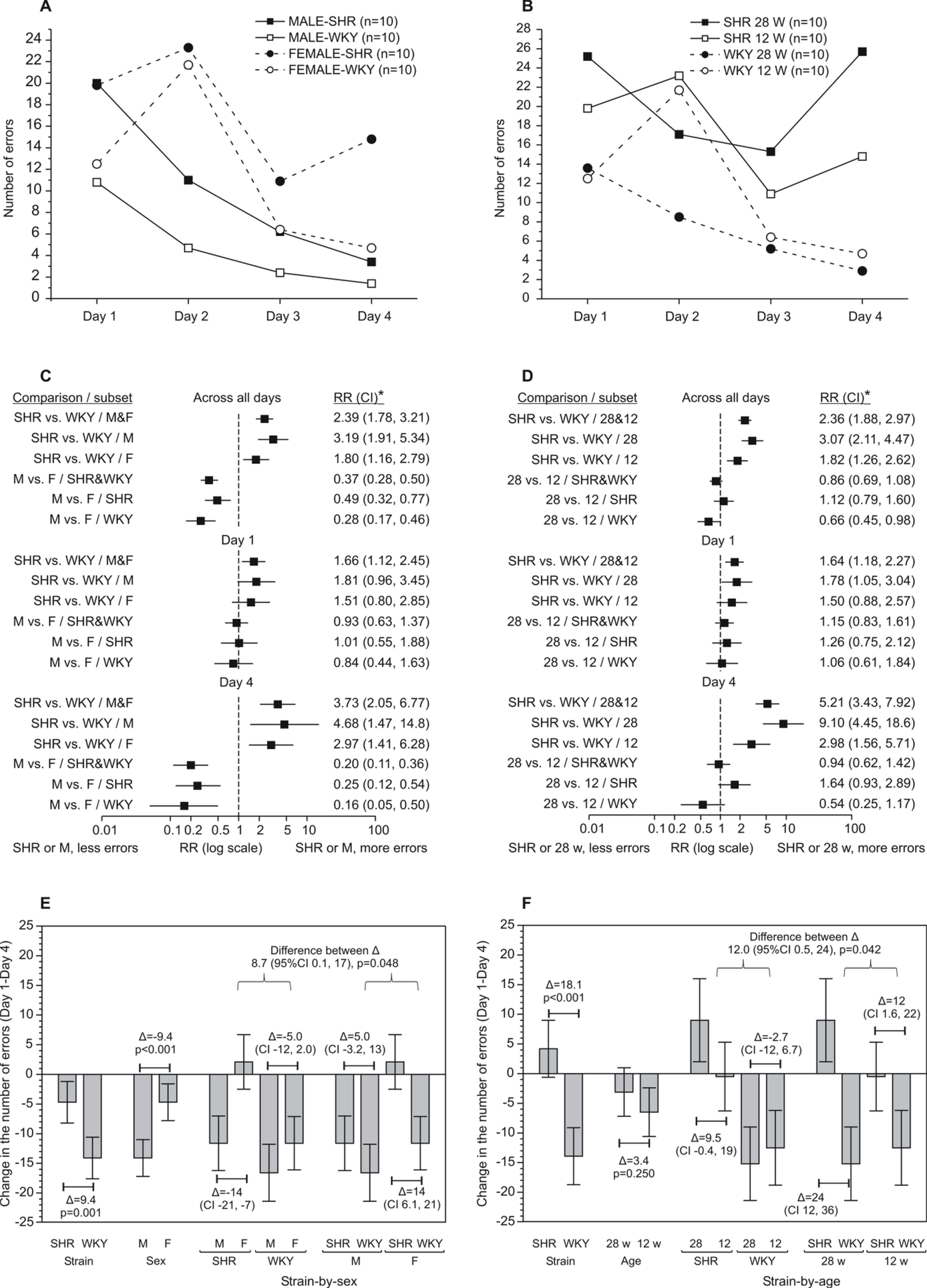


**Figure S3:** *The effect of sex and age on the cognitive performance of spontaneously hypertensive rats (SHR).* (A, C, E) 12-week old male (M) and female (F) SHR and WKY controls, (B, D, F) 28-week (7-months) and 12-week (3-months) old female SHRs and WKYs were submitted to Morris Water Maze (MWM) training trials over 4 consecutive days (10 per gender-by-strain subset). Geometric means of the cumulative number of errors (9 runs per animal) over time, by strain-by-gender (A) or by strain-by-age (B) are shown. A generalized linear mixed model (Poisson) was fitted to data from each experiment (C, D) with fixed effects strain, gender or age, time, strain*time interaction, (gender or age)*time interaction, strain*(gender or age) and strain*(gender or age)*time interaction, and with time as a repeated (random) effect. Differences between SHR and WKY overall and by gender (C) or by age (D) as well as across all days and on Day 1 and Day 4 (C, D) are shown as relative risks (RR). A general linear mixed model (E, F) was fitted to the change in the number of errors between Day 1 and Day 4 of training from each experiment with fixed effects strain, gender or age, number of errors on Day 1 and strain*(sex or age) interaction. (E) Changes by strain, by gender and by strain-by-gender subsets (data from the interaction are shown with both strain and gender as a main “plot”, for readability). Indicated are differences (Δ) between strains and between genders, overall (estimate and p-value) and by strain/gender from the interaction. Horizontal brackets in the upper part of the panel indicate a significant strain*gender interaction. (F) Changes by strain, by age and by strain-by-age subsets, data presentation as in (E). 95% Cl, 97.5Cl and 99%Cl were used, hence, when the entire RR below or above unity, p<0.05, <0.025 or <0.01.
